# Communicating climate change and health to specific target groups

**DOI:** 10.25646/11773

**Published:** 2023-11-29

**Authors:** Lena Lehrer, Lennart Hellmann, Hellen Temme, Leonie Otten, Johanna Hübenthal, Mattis Geiger, Mirjam A. Jenny, Cornelia Betsch

**Affiliations:** 1 Bernhard Nocht Institute for Tropical Medicine, Implementation Research, Health Communication Working Group, Hamburg, Germany; 2 Institute for Planetary Health Behaviour, University of Erfurt, Germany, Health Communication; 3 Institute of Psychology, University of Bamberg, Germany; 4 Harding Center for Risk Literacy, University of Potsdam, Germany

**Keywords:** PLANETARY HEALTH, COMMUNICATION, CLIMATE PROTECTION, INFORMATION BEHAVIOUR, READINESS TO ACT

## Abstract

**Background:**

The German status report on climate change and health 2023 identifies numerous health risks that are caused or exacerbated by climate change. One recommendation arising from the report is to strengthen education, information, and communication in the field. This article aims to serve as a basis for this.

**Methods:**

Based on four survey waves (2022/2023) of the PACE study (Planetary Health Action Survey, n=3,845, online), the status of risk perception as well as the Readiness to Act against climate change in the adult population in Germany is examined and a target group analysis is carried out.

**Results:**

Some health risks due to the climate crisis are perceived as comparatively low (e.g. mental health problems). People with higher risk perception show a higher Readiness to Act. Younger people, men, people with low education, and those living in smaller communities are identified as relevant target groups as they have a lower Readiness to Act. One third state that they never or hardly ever seek out specific information on climate change. Media use differs depending on target group.

**Conclusions:**

Target group-specific communication can help to educate people about the health impacts of the climate crisis. In the discussion of this article, implications from existing literature are discussed in detail, which offer practical guidance for effective climate change communication.

## 1. Introduction

### 1.1 Background

Climate change is the greatest threat to global health in the 21^st^ century [1, 2]. An overview of the health consequences can be found in the annual Lancet Countdown [3] and, with a focus on Germany, in the status report on climate change and health 2023 [4]. In Germany alone, about 150,000 deaths (due to air pollution, among other factors) could be prevented by mid-century if countermeasures were taken [5]. The health impacts particularly affect vulnerable groups such as older persons or people with pre-existing conditions [6].

The interaction of climate change and health can be summarised under the term ‘Planetary Health’ and is based on the understanding that human health depends on functioning natural systems and their sustainable use [7]. A similar concept is that of ‘One Health’, but with a strong focus on zoonoses – Planetary Health is more inclusive of climate change aspects and their interaction with human health as well as non-medical (social) determinants of health; hence the broader term is chosen here.

To minimise the health impacts of the climate crisis, countermeasures must be taken at all levels of society. This requires a significant change in people’s individual lifestyles [1, 8, 9]. However, interventions at the individual level are not sufficient [10] to deal with such a comprehensive crisis, which is why individual actions must be facilitated and accelerated by political measures [9, 11, 12]. In addition, political participation is important to initiate systemic and legislative changes [13]. These changes should be complemented and accompanied by explanatory science and risk communication to improve their effectiveness and sustainability [14].

In order to cope with health crises, risks must be perceived as such [15]. While in a German study 47% of the participants considered climate change to be dangerous [16], risk perception in relation to health has so far been rather low. In a U.S. study using a closed question format, most people stated that climate change is detrimental to health. In an open-ended question, however, only 27% of respondents were able to name specific health impacts [17]. In a German sample (representative for gender, age, education level, and federal state), it was found that people who were aware of the existence of climate change (85%) also perceived that climate change affects health (83%). However, the risk is often perceived as general rather than specific to oneself [18, 19].

These results underline the need to communicate about the health impacts of the climate crisis. The extent to which different climate change-related health effects are considered a risk can provide insights into communication gaps and opportunities. In the concluding article of the German status report on climate change and health, Mlinarić et al. [20] summarise options for action that emphasise the importance of education, information, and communication about health-related climate change risks. Knowledge and corresponding risk perception are important components of climate-friendly behavioural changes [21, 22]. Nevertheless, information, the basis for knowledge [23], is not sufficient for behavioural changes [24, 25]. In addition to knowledge, self-efficacy, trust, perceived effectiveness of measures, and social norms are relevant in determining the Readiness to Act (RTA) and should therefore be addressed in communication measures (Betsch et al.; data not published). Communication about health risks should therefore not only provide information about risks and the connection between climate change and health, but also point out options for action [18, 19]. Furthermore, for strategic communication, target groups should be identified [18, 26, 27]. This can be achieved by choosing groups with little knowledge or a low perception of health risks in order to specifically address them. Alternatively, the population can also be subdivided according to their RTA. Targeted science and risk communication can then be used to try to increase the group members’ RTA. A possible segmentation based on sociodemographic characteristics is documented in this paper. For the purposes of this study, as well as the entire PACE project (Info box), RTA is conceptualised as a combination of demonstrated individual behaviour, acceptance of potential political climate protection measures, and political participation [28]. A similar conceptualisation, based on both theoretical and empirical foundations, can be found in a publication by Stern [29] from the year 2000. Regression analyses can be used to determine which persons or groups are more or less ready to act and on which other factors this may depend. For target group analysis, the focus is primarily on sociodemographic factors (Betsch et al.; data not published).

**Info box Planetary Health Action Survey (PACE)**

**Data holder:** University of Erfurt / Bernhard Nocht Institute for Tropical Medicine

**Objective:** Based on the methods of the COVID-19 Snapshot Monitoring (COSMO), which regularly provided information on the psychological background of individual pandemic actions during the pandemic, PACE supports the handling of the climate crisis by providing the necessary social and behavioural science findings [14, 32, 33]. The surveys collect situation-specific data from questionnaires and experiments in order to identify relevant target groups as well as starting points for intervention design and climate change communication [14].

**Study design:** Repeated cross-sectional online survey (approx. 25 minutes)

**Population:** German-speaking population between 18 and 74 years of age

**Sampling:** Approx. 1,000 respondents per survey wave – representative for age, gender, and federal state according to census data, drawn by third-party provider (Acces Panel Provider: Bilendi, as of October 2023)

**Dates of data collection:** Several times per year, special surveys on particular topics (e.g. heat, nutrition) take place depending on demand.

The explorer at https://projekte.uni-erfurt.de/pace/explorer/allows an interactive analysis of the Readiness to Act and its predictors according to target groups.

More information and all questionnaires and measuring tools can be found at www.pace-studie.de.

Different media address different target groups and provide information at different levels of detail [30]. A target group analysis therefore also allows us to examine media consumption in more detail and thus identify optimal channels for reaching different groups. Media consumption varies in frequency, duration, and intensity; different media vary in accessibility, trust placed in them, and attractiveness [31]. This study more closely examines the frequency of use as well as the trust placed in different media for different target groups.

### 1.2 Overview

The German status report on climate change and health highlights numerous health risks that are caused by climate change. One recommendation arising from the status report is to provide target group-specific information about these risks. This article will serve as a basis for this by i) documenting how different health risks are perceived, ii) showing that risk perception is related to RTA on climate issues, and iii) identifying target groups with low RTA. Finally, by iv) analysing media preferences of the target groups, the ability to reach vulnerable groups and those with low RTA is shown. Recommendations for practical climate and health communication in Germany are derived from the results. In addition, the article provides an overview of existing findings on the topic and derives implications for effective climate communication in Germany from the literature that go beyond the present study results.

## 2. Methods

### 2.1 Current sample

Four dates of data collection between August 2022 and January 2023 (n1=878, n2=991, n3=960, n4=1,016) are part of this analysis. This results in a total sample of n=3,845, representative of the population in Germany by age and gender as well as federal state. The participants were invited to the study via a third-party provider and were, on average, 46 years old (standard deviation=15.24). 50% of respondents were female (n=1,933), 49% male (n=1,902); ten respondents indicated ‘diverse’ with regard to gender. The majority of respondents (55%) had completed college education with a degree (n=2,115). A tabular overview of the sample can be found in a supplement on the Open Science Framework (OSF) platform [34].

### 2.2 Constructs and measurement tools

After sociodemographic data had been collected, questions were asked about risk perception. At the first point of data collection considered in the present paper, questions were also asked about the use of and trust placed in the media. At all dates of data collection under consideration, questions were then asked about the acceptance of measures, political participation, and individual behaviour. All items and the code book can be found in the supplement [34]. The original scales and items were in German and translated to English for the purposes of this article.

#### Sociodemographic and health data

Respondents indicated several aspects of their sociodemographic status, including gender, age, state, education level, number of residents in their community, number of persons in the household, number of children under 18, employment, income, and whether they have a history of migration. The scale for income was related to the number of persons per household for further calculations [35]. In addition, the respondents were asked whether they were affected by a chronic disease.

#### Risk perception

Two components each were considered to capture risk: perceived likelihood (‘Please indicate in each case how likely these consequences of climate change are to affect your life’ – very unlikely (1) to very likely (7)) and perceived severity of various health consequences (‘…how dangerous do you think these consequences of climate change are to your life?’ – harmless (1) to extremely dangerous (7)) [36]. Perceived risk was assessed for nine health impacts triggered or exacerbated by climate change (e.g. heat, extreme weather events, increasing allergens), which are considered current threats as well as threats that will continue to increase in the future ([3, 37–39] among others). Since the perceived likelihood and severity were highly correlated (from r=0.49 to r=0.63), the two respective items per risk domain were combined into a mean value. For simpler presentation, the resulting values were reduced to three categories: low risk (1–3), medium (3.1–4.9), and high risk (5–7).

#### Information behaviour

The frequency of seeking information on climate change and the frequency of use of individual media were recorded on a 7-point scale (never (1) to very often (7)); similarly, the trust placed in individual media (very little trust (1) to very much trust (7)). The frequency of use of individual media (e.g. conversations, internet services, newspapers) and media trust were recorded once in August 2022.

#### Readiness to Act

RTA is understood as a latent, not directly measurable construct based on three indicators: individual climate protection behaviour, acceptance of political measures, and political participation. In order to find out which target groups have a low RTA, the three indicators were merged to the RTA in the overall model (Betsch et al.; data not published). The following further analyses used the resulting factor scores (see data set in supplement [34]). This means that the RTA is not a natural unit of measurement, but results from the three initial indicators at the latent level (Betsch et al.; data not published). Higher values indicate a higher RTA, i.e. more individual climate protection behaviour, higher acceptance of political measures, and more political participation. The indicators were recorded using the following scales.

##### Acceptance of political measures

The scale for the acceptance of political measures included 17 climate protection measures that were developed by the Citizen’s Assembly on Climate [40] in Germany in 2021 and presented as recommendations to politicians (Cronbach’s α=0.93). Respondents indicated their agreement with the proposals, e.g. ‘A speed limit (130 km/h) should be introduced on motorways’, from do not agree at all (1) to fully agree (7).

##### Political participation

To ensure that different forms of political participation (conventional participation as well as activism and exerting social influence) are considered, items from several existing scales [41, 42] were combined into a new scale with twelve items and transferred to climate change context (Cronbach’s α=0.93), e.g. ‘I take climate change into account when making voting decisions’, never (1) to always (7).

##### Individual behaviour

Individual behaviour was measured with the Short Impact-Based Scale of Environmental Behaviour (SIBS). The version used is an update of the measurement tool developed by Geiger, Geiger, and Wilhelm [43]. Similar to a CO_2_ calculator, it records essential areas of life and weights them according to their influence on the climate. These include housing (e.g. ‘How well insulated is your house/apartment?’, from very poor (1) to very good (7)), mobility (e.g. ‘Please state the average distance you travel by car each year as a driver or passenger’, from ‘up to 3.000 km’ (1) to ‘more than 20,000 km’ (7)), nutrition (e.g. ‘I consume all food before it spoils’, from never (1) to always (7)) and other consumption (e.g. ‘I buy particularly durable products’, from never (1) to always (7)). Subsequently, some items are recoded in such a way that a higher score indicates more climatefriendly behaviour. An index was formed from all items. The items reflect particularly CO_2_-intensive behaviour.

## 3. Results

The data analysis script and the annotated output can be found in the OSF supplement [34].

### 3.1 Risk perception

**Figure 1:**
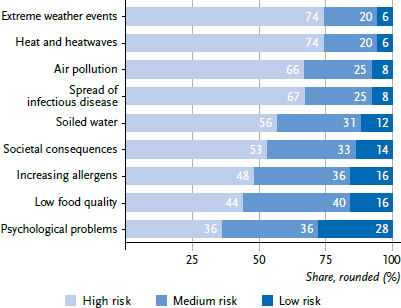
Perceived risks of various health impacts of climate change (mean of likelihood and severity per risk), data collection between August 2022 and January 2023 (n=3,845) Source: PACE 2022/2023

Figure 1 shows that the health risks of climate change were perceived very differently. Psychological consequences of the climate crisis, problems with food supply, and the increase in allergens were considered less likely and less severe. High risks were perceived for heat and extreme weather. The rankings of risks resulting from the individual assessments of likelihood and severity were very similar (see section on descriptive risk perception in the supplement [34]).

**Figure 2:**
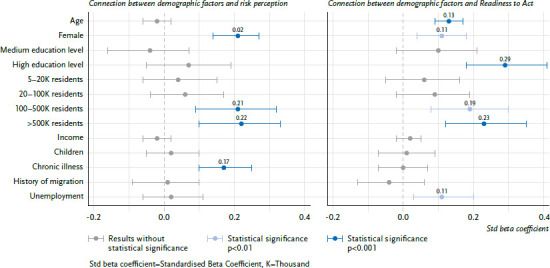
Results of the regression analyses on sociodemographic influences on risk perception (left) and Readiness to Act (right); (n=3,525) The difference to the size of the total sample is due to the exclusion of the following values: gender diverse (n=10), income not specified (n=239), chronic disease not specified (n=76), history of migration not specified (n=15). Coding: age (continuous), gender (reference: male), education (reference: low education level), residents (reference: ≤5,000), income (continuous), children (reference: no children under 18), chronic illness (reference: none), history of migration (reference: none), unemployment (reference: employment). Source: PACE 2022/2023

In order to draw conclusions about sociodemographic characteristics related to the perception of risk, a linear multiple regression on the perceived risk (mean value of likelihood and severity across all risks) was carried out (F(13, 3.511)=8.27, p<0.01). Figure 2 shows the standardised weights: women showed significantly higher risk perception than men. People residing in big cities (population >100,000) and people with chronic diseases also had a higher perception of health risks due to climate change. Of the total variance in risk perception, only a small proportion, 2.6%, can be explained by the sociodemographic factors (adjusted R^2^=0.026).

In addition, the correlation between risk perception and RTA was investigated. The two variables were strongly positively correlated (r=0.62, p<0.01) [44]. Risk perception is thus a relevant factor which, when changed, may potentially also affect the RTA.

### 3.2 Readiness to Act

The influence of sociodemographic variables was analysed in a multiple linear regression in order to identify people with low RTA as a target group for communication activities (F(13, 3.511)=10.43, p<0.01). Figure 2 shows the results (standardised beta coefficients). Of the total variance in RTA, only 3.4% could be explained by sociodemographic variables (adjusted R^2^=0.034). Younger individuals were less ready to act than older individuals and men were less ready to act than women. The largest effect was found for education: a low and medium education level was associated with lower RTA. In addition, community size was related to RTA: people living in smaller communities were less ready to act than people in larger communities. Lastly, unemployment showed a significant effect, with unemployed people having a higher RTA than those who were employed. All significant findings were small effects (ß<0.3).

In an additional regression analysis, the model was tested for possible interaction effects. The additional variance explained is so small that the effects are not considered for the present study.

### 3.3 Information behaviour

**Figure 3:**
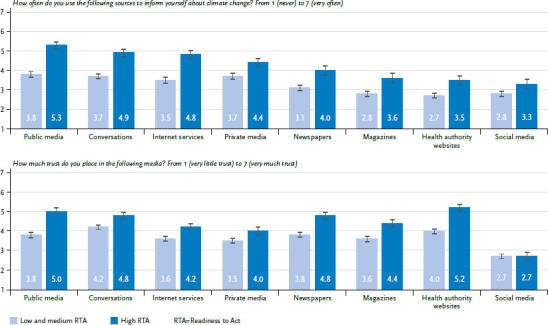
Use of and trust placed in individual media according to Readiness to Act (mean and 95% confidence intervals), survey conducted in August 2022 (n=878) Source: PACE 2022

Almost a third of the respondents said they hardly ever or never sought information about climate change (between 30–34% at the four dates of data collection), a quarter did so occasionally (26–27%) and less than half did so frequently or very frequently (39–44%). As can be seen in Figure 3, public service providers (i.e. non-privately owned media) are used most often, followed by ‘conversations with family, friends, or colleagues’. Internet services and private TV and radio stations are also used comparatively often. Social media and the websites of health authorities were used the least. In terms of trust, respondents gave the highest trust to face-to-face conversations and health authority websites, followed by public services and newspapers. Trust in social media was lowest (results also shown in the corresponding overview in the annotated output in the OSF supplement [34]).

In order to determine the groups that use individual media, linear multiple regression analyses were carried out. Here we present only a summary of the results on frequency of use; the analyses and statistical parameters on use and trust can be found in the OSF supplement [34]. Public media are primarily used by older persons. People with a high level of education are more likely to inform themselves via conversations with others than people with a low or medium level of education. Men are more likely than women to seek information via the internet, as are younger people and people with a high level of education compared to people with a low level of education. There are no significant differences in the use of private TV and radio stations. Daily and weekly newspapers are used more frequently by older persons and people with children. Whether magazines are used as a source of information about climate change depends on several variables. A larger community size (20,000 or more residents) was positively related with magazine use. Men or people with children are also more likely to use magazines. Social media are more likely to be used by younger people and people in larger communities. Health authority websites are more likely to be used by younger people, people in large cities (>500,000 residents), and people who have children.

In order to examine differences in media use among people with different levels of RTA, the sample was divided into three segments along the RTA (quantile split at ⅓ and ⅔ of the RTA). People with low and medium RTA are considered target groups here, with the greatest potential to increase understanding of the climate crisis and readiness to act against it. Therefore, they have been combined into one target group. Figure 3 shows that the two resulting groups (low to medium RTA vs. high RTA) differ significantly in frequency of use across all media types, with one consistent trend: people with high RTA generally use media more frequently than people with low and medium RTA (p<0.01 in t-tests for all media types). The largest differences are found in public media, face-to-face conversations, as well as online services. The smallest difference is found in the use of social media. For trust placed in media, the differences are also significant, with one exception: social media were considered equally untrustworthy by both groups. Once again, the group with a high RTA had higher scores overall. The largest differences can be found for trust placed in public media and newspapers as well as in health authority websites.

As mentioned in the introduction, older and chronically ill individuals are more affected by health impacts of the climate crisis than others. Therefore, the use of media was also considered according to vulnerability (for illustrations, see section on information behaviour in the OSF supplement [34]). A comparison of the risk group of older persons (65–72 years [3]) with younger people shows that older people use public media and newspapers more frequently. Online services, magazines, the websites of health authorities, and social media were used less frequently, with the difference being greatest for social media. In terms of trust, older people were found to be more sceptical about all internet services (online services, social media, health authority websites), but also about magazines and private broadcasters.

A comparison of the chronically ill with the non-chronically ill showed few significant differences. The chronically ill used social media, health authority websites, and magazines slightly less often. Trust only differed for social media, where the chronically ill had lower scores.

## 4. Discussion

### 4.1 Summary and discussion of the results

The results of this evaluation over four waves of the PACE study (Info box) show that some health risks due to the climate crisis are perceived as comparatively low, e.g. mental health problems, even though the climate change-related increase is already posing a challenge [45]. People with greater risk perception show a higher RTA. Even if no causal statements are possible, theoretical models [46] and empirical findings [21, 22, 47] indicate that education about health risks can lead to an increase in general RTA. Our results on risk perception correspond to those of similar studies [19]. Risks that are obvious, discussed in the media, and possibly also experienced, such as heat and heatwaves, are perceived as greater than those that are less obvious or less frequently discussed in the media (e.g. mental health problems). Even if it is not clear what the ‘correct’ response on the given scale would be in the current situation, these results show a certain discrepancy with the real health threats posed by the climate crisis, as also noted in the status report on climate change and health [4]. This can be illustrated by the example of pollen count: although increasing problems such as respiratory complaints and diseases due to increased allergen exposure have been recorded for years [38, 39], almost one third of the respondents consider the risk (likelihood, severity) to be low.

When segmenting the target groups based on sociodemographic variables, younger people, men, people with a low level of education, and those living in smaller communities show up as relevant target groups for climate and health communication. However, it is very important to note that only a very small proportion of the variance (3.4%) of the RTA can be explained by the sociodemographic factors mentioned. Thus, although this analysis is suitable for determining target groups, further and different variables must be added to achieve an understanding of the differences (e.g. risk perception, social norms, self-efficacy [26, 48, 49]).

The presented results on age may seem remarkable and perhaps counterintuitive to some: older people show higher RTA values than younger people. This contradicts some earlier research findings [50] as well as a subjective impression of informed and committed young people, which may have arisen through media reports since the beginning of the protests and school strikes led by Fridays for Future [51]. It must be noted here that only people aged 18 and older took part in this survey; the school strikes, however, are mainly attended by younger people [52] – no statements can be made about the underage group here. Frustration with political processes as well as a lack of trust and the resulting lack of self-efficacy may explain somewhat lower acceptance of political measures and generally rather low political participation [50]. However, the exact correlations are not clear. An overriding factor could also be that there are a large number of issues competing for young people’s attention. It is possible that those they feel more directly affected by are prioritised, such as job prospects, the economic situation, or education [50]. Climate issues may thus receive less attention and lower priority in everyday actions. A comparison of a subset of items with individual items from other surveys on the same topic [53] shows that other studies document similar tendencies as this study. For example, although older people’s residences tend to be larger, as is reflected in the present data, they also state, e.g., that they take shorter showers than younger people, while hardly any differences are shown in overall dietary behaviour. When it comes to mobility and the purchase of clothing, an inverted U-shape was found, with young and older persons in particular showing climate-friendly behaviour [54]. For individual behaviour, the present study only asked about behaviours with a major social impact on the climate that are carried out by the majority of people (e.g. not taking a sauna, an activity where older persons show more climate-damaging behaviour) and that have a relevant overall impact on the consumption of fossil energy (e.g. not the comparatively low electricity consumption through lighting). For the overall index, the behaviours were additionally weighted according to their degree of impact. This specific selection and the weighting could have contributed to the differences between age groups. Further research is needed to examine the above-mentioned correlations for Germany in more detail. Looking at the RTA according to its individual components can contribute to this (Lehrer et al.; data not published).

The (mass) media play a significant role in building up knowledge about the risks of climate change [55]. Just under a third of respondents stated that they seek little or no specific information. In particular, people with a low RTA seek information less than people with a high RTA. Thus, the people who may need the information the most seem to be the most difficult to reach – because they place less trust in the various available types of media for climate reporting and education, amongst other reasons. It therefore seems prudent to integrate this topic in those media and media formats that are also used by people with a low RTA (public media, conversations with trusted individuals, internet services, private broadcasters; see Figure 3). Concerning the specific sources, the websites of the health authorities stand out. Despite the high level of trust placed in them, they are the least used of all the media surveyed, which indicates accessibility issues and does not earmark them as media that should be relied on in the future – at least not without corresponding publicity campaigns. Vulnerable groups (here: older and chronically ill persons) show only few specific and rather unsurprising media preferences. The fact that older persons use classical media (public broadcasters and newspapers) more and online services less is consistent with earlier findings [56]. Communicators should always start with the question of how the information reaches the target group and not just rely on people actively looking for it.

### 4.2 Methodological limitations of the study

It must be noted that there are other vulnerable groups that were not explicitly part of this study or whose characteristics were not assessed. These include people residing in areas particularly at risk (e.g. coastal regions), groups with low socioeconomic status, children, as well as pregnant and breastfeeding women [6].

Furthermore, the online format of the survey as well as the recruitment of the sample via a third-party provider lead to limitations despite the intended representativeness. The present sample has an above-average level of education and, especially for the older group, a bias with regard to lifestyle and attitudes is to be expected, since not all people are connected to the internet at an older age. Due to the monetary incentive, it is possible that a fast and thus possibly unfocused response was a priority for the participants, which could be detrimental to the data quality. In general, data quality is difficult to ensure in online surveys because it is not known how attentive participants are. With regard to the results, it is important to emphasise the small effect sizes of the sociodemographic influences on risk perception and RTA. No patterns emerged for media use, which may also be due to the fact that media types were surveyed rather than specific media (e.g. individual channels or apps). A more distinct differentiation could provide detailed insights in the future.

### 4.3 Previous research findings and practical implications

In the following part of the discussion, we take a broader view: based on further literature on the topic, implications for effective climate communication in Germany are derived that go beyond the present study results.

#### Risk perception, emotions, and self-efficacy

As explained above, the starting point of any crisis behaviour is the perception of a threat. In most models of crisis and health behaviour, risk perception is the core (e.g. Protection Motivation Theory (PMT) by Rogers [57], Health Belief Model by Rosenstock [58]). Without the perception of a threat, action against the climate crisis is very unlikely. Messages on the climate crisis should therefore include the presentation of risks. The results considered here can provide indications as to which risks require special education. However, risk perception and the resulting decisions are not only evolving from the transfer of information, but also by the emotions associated with it [59]. Emotionality is an important component of communication, which can initiate changes in behaviour by triggering concern [60]. However, this initially apparently positive effect has limits: if an audience is repeatedly confronted with frightening images of a possible future, interest in the problem decreases, those affected experience a state of topic-specific fatigue, refuse to absorb new information [61] or feel reactance (anger [62]), and are stimulated to behave in a contrary way [63]. In relation to the climate crisis, for example, the terms ‘apocalypse fatigue’ [64] or ‘climate fatigue’ [65] are used. Negative or threatening messages in climate change communication must therefore be used with caution in order to avoid undesired effects [66]. These assumptions are clearly formulated in PMT [46], which assumes that fear appeals without clear instructions on how to increase self-efficacy and avert the threat lead people to fall into a state of denial and resistance. It can be deduced from this that negative emotions can only be used effectively in communication if they are accompanied by a self-efficacy message [67]. One helpful approach are stories that show how climate protection and solutions can be a part of real life situations, with positive emotional connotation. However, even purely positive or optimistic portrayals have their pitfalls. They can lead to people being less willing to commit to climate protection. Communication that triggers moderate fear can therefore be helpful, as long as it presents risks realistically [68].

Theoretically [48] and empirically [69], the identification of possibilities for action and the resulting self-efficacy are associated with a greater RTA and are strongly recommended. Self-efficacy refers to people’s confidence in their own potential and competencies to effectively cope with demands – i.e. ‘I can if I want to’ [70]. According to Bandura [71], there are several ways in which self-efficacy can be promoted. In addition to one’s own experiences, this also occurs through vicarious experiences (observation), emotions, and instructions. One’s own experiences are considered the most effective option and can be supported by events, trainings, or stories. Case studies and the presentation of people or events with a role model character can help with communication. The greater the identification with the person used, the higher their impact [70]. Cinematic portrayals of successful climate behaviour, therefore, are one way to increase self-efficacy through observational learning, emotion, and identification.

#### Health as a co-benefit

Strategic framing of information can be used as another approach in climate change communication [72]. This involves drawing attention to individual contexts and sub-facets of a topic, which affects how information is understood and categorised [73, 74]. Health as a frame for climate change issues has the potential to increase the personal relevance of the issue and shift the perception of the issue from a distance directly into the lives of the people receiving the information [75]. Health can thus help to reduce the psychological distance to the climate issue and at the same time increase the subjective importance of the issue [76]. Among people who are not concerned about the climate crisis, health framing of climate action was shown to be particularly effective [72]. Health framing has potential in future climate communication, as it has been used comparatively infrequently so far and thus represents an alternative to the much-communicated environmental harm frame [77, 78]. While the results of some studies clearly speak for the use of health frames, others, however, cannot find any difference to other frames or even find a negative effect [79, 80]. Possible reasons for these heterogeneous results are being investigated in more detail in the PACE study (Lehrer et al.; data not published). Another advantage that arises from a focus on health consequences of the climate crisis is the chance to tap into new communication channels. For example, risk communication by health professionals can be one way to reduce rejection of climate action [19]. Everyday barriers stand in the way of this solution, but these could be overcome through additional resources (e.g. further education and communication training for professionals, materials for patient education) [81]. This is also recommended in the status report on climate change and health [4].

#### Interactive communication

One way to achieve interactive and participatory knowledge transfer is for well-known people from a community to share knowledge within their own community. This type of exchange can be more accessible and trustworthy than messages from people with whom there is no personal connection, e.g. politicians [24, 82, 83]. Therefore, it is important to empower different people from various backgrounds to act as ‘climate champions’ for climate protection. The principle of such champions has been researched in the field of vaccination, for example [82, 84, 85]. Vaccine champions are successful because they shape the social norm and can thus influence behaviour. We are more likely to trust people who are similar to us [86] or who we believe represent us well [82]. Similarly, climate champions could steer behaviour in their social environment, e.g. at work, in volunteer settings, or in clubs and societies, towards climate protection. They can share their own experiences, disseminate knowledge through reputable sources, and lend a sympathetic ear for questions or concerns. Target groups that seek information mainly through conversations or social media can likely be reached through such formats.

Another possibility for interactive knowledge transfer is to link it to other interests. Creative and often emotional formats of knowledge transfer are needed, for example incorporating art or music [87]. At such events, interested people can exchange ideas with researchers and other experts. The combination of food and drink in the form of a knowledge buffet or café is also a promising approach. This creates a positive atmosphere – one study showed that we are more likely to agree to speeches if we eat while listening [88]. In a knowledge buffet on the connection between climate and nutrition, a student target group showed a clear increase in knowledge as well as higher self-efficacy with regard to climate-friendly nutrition (Otten et al.; data not published).

#### Knowledge transfer alone is not enough

Knowledge transfer alone cannot initiate social change [24, 89] and usually cannot trigger the necessary level of action. Scientists speak of various challenges in planetary health education: in addition to the knowledge challenge, there is also the imagination challenge [90] and the implementation challenge [7, 91]. This is in line with what was said about communication at the beginning of this paper: communication must include more than just knowledge [18, 19]. While informing individuals about individual actions and activities is undoubtedly important, educational offers for (potential) ‘change agents’ (designers of transformation processes [91]) or the climate champions discussed above can be helpful and have a multiplication effect [91]. Positive emotions play an important role in education on climate change and health [92, 93]. Positive visions and ways of communicating can strengthen hope, which is considered the most important emotion in this context and can overcome feelings of fear, among other things [92]. In general, positive emotions can have a favourable effect on dealing productively with the climate crisis [94]. Research on framing has also shown that a presentation of possibilities and opportunities of climate protection instead of perceived losses can be useful, especially among less concerned people [72].

#### Coordination of communication and policy measures

According to a meta-analysis, campaigns in the health sector were more likely to persuade their target group to change their behaviour if political or legal changes favoured this change [95]. Political measures are essential for fast and effective climate protection anyway [96]. This reinforces the relevance of looking at more than individual behavioural change and directs attention to the conditions under which policy measures are accepted [97, 98] and how political participation can be strengthened. Both aspects were considered to be essential parts of the RTA in the present study. Social change needs communication. Conversely, however, it is also true that the best communication does not help if structural change does not occur [99]. This is also reflected in the overarching options for action of the status report on climate change and health, which calls for the connection of communicative measures to measures of structural prevention [20].

## 5. Conclusion

Overall, this article shows that there are some relevant target groups in Germany who perceive climate change-related health risks to be low and who have a low RTA against climate change. Even if these can be identified by statistical methods, the differences between target groups and people who do not belong to the target groups are relatively small. This means that while demographically defined target groups do have specific needs or touchpoints (e.g. media channels), addressing the population as a whole is another important task. For this purpose, it can be helpful to institutionalise communication about climate and health [100]. Existing structures for education and health promotion could be used, which would be a sensible approach considering the high level of trust in the health sector. The available data reflect this through the high level of trust placed in associated media (health authority websites), albeit with a relatively low level of use. Targeted campaigns that draw attention to offers specific to the target group can help to change this. Non-intended effects of communication (such as weakening of the RTA, reactance) should be anticipated and prevented as far as possible [63]. Options for action and positive emotions should play a role; risks should be communicated together with messages of effectiveness.

For preparing targeted communication, an interactive explorer is available on the website of the PACE study [101], in which up-to-date results can be displayed interactively for different target groups over time. In addition, further variables can also be analysed over different points in time and viewed grouped according to certain characteristics. Depending on interest and need for intervention, possible starting points can thus be identified. This tool can support effective, target group-specific climate and health communication in Germany.

## Key statement

- The perception of climate change-related health risks is an important correlate of Readiness to Act.
- Some climate change-related health risks are perceived as lower than others by the general population (e.g. mental health problems, low food quality, or the increase of allergens).
- Younger people, men, people with low education, and those living in smaller communities are more likely to have a low Readiness to Act.
- Only a small extent of one’s Readiness to Act against climate change is determined by sociodemographic influences.
- People with low Readiness to Act are less likely to seek information independent from media type, which makes it particularly difficult to reach them.
- The explorer at www.pace-studie.de allows an interactive analysis of the Readiness to Act and its influencing factors according to target groups.
